# Supplementary material for: Conformational changes of the baseplate regulating tail contraction of Staphylococcus phage 812
Source: EMBO J. 2026 Jun 26;45(15):5514–36. doi: 10.1038/s44318-026-00834-9 (PMC13433997; doi:10.1038/s44318-026-00834-9)
Supplement: Supplementary file 2 [file 44318_2026_834_MOESM2_ESM.pdf]

THE EMBO JOURNAL

Conformational changes of the baseplate regulating tail contraction of Staphylococcus phage 812

Ján Bíňovský, Marta Šiborová, Maryna Zlatohurska, Jiří Nováček, Pavol Bárdy, Roman Baška, Karel Škubník, Tibor Botka, Martin Benešík, Roman Pantůček, Konstantinos Tripsianes, and Pavel Plevka

Corresponding author(s): Pavel Plevka (pavel.plevka@ceitec.muni.cz)

Review Timeline:

Submission Date:	4th Feb 25
Editorial Decision:	19th Mar 25
Revision Received:	29th Apr 25
Editorial Decision:	30th May 25
Appeal Received:	8th Mar 26
Editorial Decision:	14th Apr 26
Revision Received:	25th May 26
Accepted:	29th May 26

Editor: Hartmut Vodermaier

Transaction Report:

(Note: With the exception of the correction of typographical or spelling errors that could be a source of ambiguity, letters and reports are not edited. Depending on transfer agreements, referee reports obtained elsewhere may or may not be included in this compilation. Referee reports are anonymous unless the Referee chooses to sign their reports.)

1st Editorial Decision **19th Mar 2025**

Dr. Pavel Plevka
CEITEC, Masaryk University
Structural virology
Kamenice 5/E35
Brno 62500
Czech Republic

19th Mar 2025

Re: EMBOJ-2025-120378
Conformational changes of baseplate linked to tail contraction of S. aureus phage phi812

Dear Dr. Plevka,

Thank you for submitting your structural study on baseplate and tail dynamics during phage phi812 infection of S. aureus. We have now received reports from three expert referees, copied below for your information. As you will see, they all appreciate the novel insights into infection of a cell-wall-bearing Gram-positive bacterium. However, referee 2 raises a number of substantive concerns with the structural analyses and atomic modeling, especially in light of the obtained resolution, which would need to be satisfactorily addressed prior to publication.

Should you be able to adequately address these criticisms (as well as the various other, more specific points), we would be happy to consider a revised version further for The EMBO Journal. Please be reminded, however, that our single-major-revision-round policy makes it important to diligently respond to each referee point at the time of resubmission; therefore, please do not hesitate to contact me ahead of resubmission with any questions you may have in this regard already. We would also be open to extension of the regular three-months revision period if needed; our 'scooping protection' (meaning that competing work appearing elsewhere in the meantime will not affect our considerations of your study) would of course remain valid also throughout such an extension.

Further information on preparing, formatting and uploading a revised manuscript can be found below and in our Guide to Authors. Thank you again for the opportunity to consider this work for The EMBO Journal, and I look forward to receiving your revised manuscript in due time.

Yours sincerely,

Hartmut Vodermaier

Hartmut Vodermaier, PhD
Senior Editor, The EMBO Journal
h.vodermaier@embojournal.org

*** PLEASE NOTE: All revised manuscript are subject to initial checks for completeness and adherence to our formatting guidelines. Revisions may be returned to the authors and delayed in their editorial re-evaluation if they fail to comply to the following requirements (see also our Guide to Authors for further information):

1) Every manuscript requires a Data Availability section (even if only stating that no deposited datasets are included). Primary datasets or computer code produced in the current study have to be deposited in appropriate public repositories prior to resubmission, and reviewer access details provided in case that public access is not yet allowed. Further information: embopress.org/page/journal/14602075/authorguide#dataavailability

2) Each figure legend must specify
- size of the scale bars that are mandatory for all micrograph panels
- the statistical test used to generate error bars and P-values
- the type error bars (e.g., S.E.M., S.D.)
- the number (n) and nature (biological or technical replicate) of independent experiments underlying each data point
- Figures may not include error bars for experiments with n<3; scatter plots showing individual data points should be used instead.

3) Revised manuscript text (including main tables, and figure legends for main and EV figures) has to be submitted as editable text file (e.g., .docx format). We encourage highlighting of changes (e.g., via text color) for the referees' reference.

4) Each main and each Expanded View (EV) figure should be uploaded as individual production-quality files (preferably in .eps, .tif, .jpg formats). For suggestions on figure preparation/layout, please refer to our Figure Preparation Guidelines: http://bit.ly/EMBOPressFigurePreparationGuideline

5) Point-by-point response letters should include the original referee comments in full together with your detailed responses to them (and to specific editor requests if applicable), and also be uploaded as editable (e.g., .docx) text files.

6) Please complete our Author Checklist, and make sure that information entered into the checklist is also reflected in the manuscript; the checklist will be available to readers as part of the Review Process File. A download link is found at the top of our Guide to Authors: embopress.org/page/journal/14602075/authorguide

7) All authors listed as (co-)corresponding need to deposit, in their respective author profiles in our submission system, a unique ORCiD identifier linked to their name. Please see our Guide to Authors for detailed instructions.

8) Please note that supplementary information at EMBO Press has been superseded by the 'Expanded View' for inclusion of additional figures, tables, movies or datasets; with up to five EV Figures being typeset and directly accessible in the HTML version of the article. For details and guidance, please refer to: embopress.org/page/journal/14602075/authorguide#expandedview

9) To facilitate reproducibilty and cross-laboratory adoption of methodologies, please structure the Materials & Methods section as outlined in our guide to authors, including a completed Reagents and Tools Table that can be downloaded from our author guidelines as well (https://www.embopress.org/page/journal/14602075/authorguide#structuredmethods).

10) Digital image enhancement is acceptable practice, as long as it accurately represents the original data and conforms to community standards. If a figure has been subjected to significant electronic manipulation, this must be clearly noted in the figure legend and/or the 'Materials and Methods' section. The editors reserve the right to request original versions of figures and the original images that were used to assemble the figure. Finally, we generally encourage uploading of numerical as well as gel/blot image source data; for details see: embopress.org/page/journal/14602075/authorguide#sourcedata

At EMBO Press, we ask authors to provide source data for the main manuscript figures. Our source data coordinator will contact you to discuss which figure panels we would need source data for and will also provide you with helpful tips on how to upload and organize the files.

Further information is available in our Guide For Authors: https://www.embopress.org/page/journal/14602075/authorguide

In the interest of ensuring the conceptual advance provided by the work, we recommend submitting a revision within 3 months (17th Jun 2025). Please discuss the revision progress ahead of this time with the editor if you require more time to complete the revisions. Use the link below to submit your revision:

Link Not Available

Referee #1:

This paper presents a comprehensive structural analysis of the events that occur during bacteriophage phi812 infection of Gram-positive Staphylococcus aureus. The study provides high-resolution insights into the sequence of conformational changes that govern baseplate restructuring, tail contraction, and subsequent genome delivery into the bacterial cytoplasm. While similar studies have been conducted for bacteriophages infecting Gram-negative bacteria, the structural details of phage-host interactions in Gram-positive bacteria, which possess a markedly different cell wall architecture, remain much less understood owing to the lack of high-resolution structural information. As such, this study represents a significant step forward in bridging this knowledge gap and will serve as a foundational reference for future research into bacterial infection and phage-host interactions.

I suggest the following changes:

1)Abstract: The authors should explicitly highlight the key experimental techniques underpinning their findings, particularly cryo-EM and cryo-ET, and emphasize how their results advance our broader understanding of phage infection mechanisms.

2)While the study emphasises the novelty of characterising a Gram-positive phage, it would be valuable to more explicitly discuss the similarities and differences between genome delivery mechanisms in Gram-positive and Gram-negative bacteria. Some differences are mentioned in passing in several sections, but it would be valuable to summarise these either in a separate section or in the Conclusions.

Minor points:

1) Some of the text in the figures (e.g., Fig. 1) is difficult to read due to the too small font size. Could this be increased for better readability?

2) Tables S1, S4. Several values are reported with excessive precision. Please round B-factors to one decimal place, RMS bond angles to one or two decimal places, and Ramachandran outliers to one decimal place for meaningful accuracy.

Referee #2:

This study presents a comprehensive structural and modeling analysis of phage 812 baseplate composition and conformational dynamics upon sheath contraction. The study is significant in two respects.
First, it provides initial atomic models of all baseplate building blocks (e.g., core, wedge modules, baseplate arms, and tripod complexes) of a large Myoviridae phage infecting a Gram-positive bacterium. These models are primarily predicted and restrained against medium/low-resolution densities (at least for the native baseplate), nonetheless obtaining a level of accuracy greater than anything done so far for a gram-positive phage. Second, the authors observed and described an unexpected rigidification and symmetrization of the baseplate upon sheath contraction, allowing them to impose C6 and generate realistic atomic models. Based on these findings, they propose a rather basic model for cell wall degradation and possibly genome ejection.
Overall, I think the paper has merit and provides a significant advancement in the field of gram-positive phages. However, I am not convinced that the extensive modeling used to build atomic models aggressively at low resolution is fully validated and backed up by additional confirmatory experiments. My most significant concern is that the authors do not have the resolution to fit a structural atlas into their reconstructions except for the C6 maps, which are near-atomic resolution. Without functional validation by MS and genetic knockouts, it isn't easy to assess the validity of these models.

Major comments

1 - Provide a Table with all the polypeptide chains identified by Mass Spectrometry (e.g., number of hits, significance, MW, etc.) for all phage specimens utilized in this study. Given the low resolution of most reconstructions presented here, the authors must validate the existence of all AlphaFold models docked in the density with MS hits. Without this effort, the paper lacks rigor.

2 - Reconstruction of the Native baseplate. The resolution is low, and the FSC curves in Fig S3 look very poor at low res, suggesting multiple conformations have been averaged together. The authors should explain the poor correlation at low resolution. Also, can these maps be modeled with enough accuracy? How low is too low to place an AlphaFold model into the density?
3 - Phage 812 head, neck and tail. What happened to the rest of the phage? Why keep this data out of this work?

4 - Cryo-EM refinement. How were the many models presented in this study refined against the various cryo-EM maps? Again, how rigorous is this cryo-EM analysis? Structural refinement must be described in the methods. For all models presented in this study, include a Model-to-Map Correlation Coefficient in the Table, which is the closest to a Rfree in cryo-EM. Without this, the paper lacks rigor.

5 - What do the authors mean by 'Surface representations of cryo-EM reconstructions"? Are they showing an electron density map or the solvent surface obtained from a model built into a map?
If the former, they should state the resolution of each map shown in the figures (at a given FSC cut-off) and the relative contour at which the map is displayed.

6 - Central Spike. It is unclear reading this paper if the authors see this protein or just modeled it.
On line 141, they call Fig 2B, which does not show a central spike '(Fig. 2B, SFig. 1, 2J, 3, Table S1)').
Is this a mistake? In fig S2J they show the recombinant trimeric knob. But what about the coiled-coil? Was this feature ever observed? How can a reader tell if the figure shows an experimental model of pure fantasy? At times, the paper comes across as a modeling exercise.

7 - Fig. 7. Why is the inner membrane of gram-positive labeled as OM in the diagram? Shouldn't this be a plasma membrane or an Inner Membrane (IM)? Also, what is the evidence to show that the entire tail tube penetrates the IM? Such a model is unlikely. The author should provide evidence or at least cite literature that the tail tube crossed the IM and dipped into the bacterium cytoplasm. Again, this seems odd.

8 - Table S4. X-ray data and structure quality indicators should be renamed 'X-ray data collection and refinement statistics'. Also, include the reflections in the outer shell and all relative statistics for the outer shells.

9 - NMR studies: why did the authors use NMR to solve the cleaver domain? It's a globular and easy-to-predict domain that AlphaFold can likely get with 99% accuracy

Referee #3:

1/ The manuscript describes - to my knowledge- the first myophage infection a monoderm, as most monoderm (Firmicutes and Actinobacteria) infecting phages are siphophages and in a lesser extent podophages. This phage possess RBPs that target polysaccharides of host, and not proteinaceous receptors. Furthermore, the structure is of high quality,and, when some elements of the phages were not visible due to stistic disorder, they were predicted by AlphaFold2. Both rest and activated structure have been determined, making it possible to elaborate on the infection mechanism. The paper is well written and equisitely illustrated.
- There is no major concern.
- Minor concerns:a) Figure 2E, assembly (not assebly)
b) In Figure 7, the RBP2 is pointing to the host cell wall (as in Figure 6C) and RBP1 is pointing to the side, which is strange for host binding). Furthermore, RBP1 is not visible in Figure6. Could you explain?
- Suggestions: In most recent structures (of siphophages mainly), the C-terminus of the TMP is visible. Did you see any density (helical probably) that could be assigned to the TMP? In any case, that should be commented.

Referee comments are in blue italics, **and our responses are in black bold font. Please note that the indicated line numbers refer to the manuscript pdf with tracked changes.**

Referee #1:

This paper presents a comprehensive structural analysis of the events that occur during bacteriophage phi812 infection of Gram-positive Staphylococcus aureus. The study provides high-resolution insights into the sequence of conformational changes that govern baseplate restructuring, tail contraction, and subsequent genome delivery into the bacterial cytoplasm. While similar studies have been conducted for bacteriophages infecting Gram-negative bacteria, the structural details of phage-host interactions in Gram-positive bacteria, which possess a markedly different cell wall architecture, remain much less understood owing to the lack of high-resolution structural information. As such, this study represents a significant step forward in bridging this knowledge gap and will serve as a foundational reference for future research into bacterial infection and phage-host interactions.

I suggest the following changes:

1)Abstract: The authors should explicitly highlight the key experimental techniques underpinning their findings, particularly cryo-EM and cryo-ET, and emphasize how their results advance our broader understanding of phage infection mechanisms.

R: We have now modified the abstract by including information about the use of cryo-EM and a summary sentence:

Lines 23-25: "Here, we used cryo-EM to show that the baseplate of phage phi812, which infects Gram-positive Staphylococcus aureus, is formed of a core, wedge modules, and baseplate arms carrying receptor-binding proteins type 1 and 2 and tripod complexes."

Lines 32-33: "Homologous molecular mechanisms are probably shared by phages with contractile tails to infect Gram-positive bacteria."

2)While the study emphasises the novelty of characterising a Gram-positive phage, it would be valuable to more explicitly discuss the similarities and differences between genome delivery mechanisms in Gram-positive and Gram-negative bacteria. Some differences are mentioned in passing in several sections, but it would be valuable to summarise these either in a separate section or in the Conclusions.

R: Thank you. We have now extended the discussion of the similarities and differences between genome delivery mechanisms of phages infecting Gram-positive and Gram-negative bacteria in the Conclusions paragraph:

Lines 711-712: "In contrast, phages employ central spike proteins with rigid β-prism or β-helix domains to penetrate the outer membrane of Gram-negative bacteria 41–43."

Lines 717-720: "The structures of the baseplate core, tail sheath, and tail tube proteins of phi812 are similar to those of phages infecting Gram-negative bacteria41–43, indicating that the mechanism triggering the tail sheath contraction is conserved among the phages infecting hosts with distinct cell wall properties."

Lines 722-727: "The tail sheath shortens from 200 to 96 nm upon contraction23, pushing the tail tube's tip through the peptidoglycan layer and plasma membrane 10-30 nm into the cytoplasm, depending on the local thickness of the S. aureus cell wall. The hub proteins with putative peptidoglycan-degrading activity are positioned at the tip of the phi812 tail tube and may facilitate its penetration through the cell wall. In contrast, T4 baseplate lysozymes are released from the baseplate into the periplasmic peptidoglycan layer of Gram-negative E. coli8,33."

Minor points:

1) Some of the text in the figures (e.g., Fig. 1) is difficult to read due to the too small font size. Could this be increased for better readability?

R: We have now increased the font size in all main figures and some supplementary figures.

2) Tables S1, S4. Several values are reported with excessive precision. Please round B-factors to one decimal place, RMS bond angles to one or two decimal places, and Ramachandran outliers to one decimal place for meaningful accuracy.

R: We have now modified the tables as requested.

Referee #2:

This study presents a comprehensive structural and modeling analysis of phage 812 baseplate composition and conformational dynamics upon sheath contraction. The study is significant in two respects.
First, it provides initial atomic models of all baseplate building blocks (e.g., core, wedge modules, baseplate arms, and tripod complexes) of a large Myoviridae phage infecting a Gram-positive bacterium. These models are primarily predicted and restrained against medium/low-resolution densities (at least for the native baseplate), nonetheless obtaining a level of accuracy greater than anything done so far for a gram-positive phage. Second, the authors observed and described an unexpected rigidification and symmetrization of the baseplate upon sheath contraction, allowing them to impose C6 and generate realistic atomic models. Based on these findings, they propose a rather basic model for cell wall degradation and possibly genome ejection.
Overall, I think the paper has merit and provides a significant advancement in the field of gram-positive phages. However, I am not convinced that the extensive modeling used to build atomic models aggressively at low resolution is fully validated and backed up by additional confirmatory experiments. My most significant concern is that the authors do not have the resolution to fit a structural atlas into their reconstructions except for the C6 maps, which are near-atomic resolution. Without functional validation by MS and genetic knockouts, it isn't easy to assess the validity of these models.

Major comments

1 - Provide a Table with all the polypeptide chains identified by Mass Spectrometry (e.g., number of hits, significance, MW, etc.) for all phage specimens utilized in this study. Given the low resolution of most reconstructions presented here, the authors must validate the existence of all AlphaFold models docked in the density with MS hits. Without this effort, the paper lacks rigor.
R: We have included a new Appendix Table S2 with the requested MS analysis.

2 - Reconstruction of the Native baseplate. The resolution is low, and the FSC curves in Fig S3 look very poor at low res, suggesting multiple conformations have been averaged together. The authors should explain the poor correlation at low resolution. Also, can these maps be modeled with enough accuracy? How low is too low to place an AlphaFold model into the density?
R: We agree with reviewer #2 that the resolution of the reconstruction of the native baseplate is limited, which may be due to the averaging of multiple conformations, even though the reconstruction process included 2D and 3D classifications. We have now included this explanation in the manuscript (Lines 1462-1470):
"The resulting map of the baseplate reached a resolution of 7.3 Å after the post-processing, with a calibrated pixel size of 1.075 Å/px applied. The limited resolution of the reconstruction may be due to the variability in the structures of the individual baseplates, which results in the averaging of multiple conformations in the reconstruction, even though the reconstruction process included 2D and 3D classifications. To obtain a higher resolution structure of the central part of the tail that is less flexible, alternating rounds of 3D auto-refinement and CTF refinement were performed using a mask including the first three tail sheath rings, core, and wedge proteins, yielding a map with a resolution of 4.5 Å and a calibrated pixel size of 1.075 Å/px."

To demonstrate that the maps can be modeled with adequate accuracy, we have now included a new supplementary figure (SFig. 4) showing proteins forming the lower and upper baseplate arms fitted into their respective regions of the cryo-EM map. For each chain, we provided Cross-Correlation (CC) values calculated using the *Comprehensive Validation (cryoEM)* tool from the Phenix software package. Resolution at which accurate placement of a predicted model into a

low-resolution cryo-EM density is possible cannot be generalized because it depends on the presence of specific structural features in the macromolecular model and map. The images shown in Sfig. 4 enable validation of the quality of the structure fitting into the reconstruction of the native baseplate.

3 - Phage 812 head, neck and tail. What happened to the rest of the phage? Why keep this data out of this work?

R: We are preparing another manuscript describing the structure of phi812 head and neck. The combined manuscript would be too long.

4 - Cryo-EM refinement. How were the many models presented in this study refined against the various cryo-EM maps? Again, how rigorous is this cryo-EM analysis? Structural refinement must be described in the methods. For all models presented in this study, include a Model-to-Map Correlation Coefficient in the Table, which is the closest to a Rfree in cryo-EM. Without this, the paper lacks rigor.

R: We have now included Table S6, summarizing the refinement procedures used for the different reconstructions and their protein components. The table is referenced in the Materials and Methods section (Lines 1742-1743):
"Refinement strategies were selected based on the resolution and symmetry of the available maps (Appendix Table S6)."

We have now added Model-to-Map Correlation Coefficients for all the structures in Appendix Table S1. Please note that Map-to-Model FSC curves are provided in Appendix Figure 3.

5 - What do the authors mean by 'Surface representations of cryo-EM reconstructions"? Are they showing an electron density map or the solvent surface obtained from a model built into a map? If the former, they should state the resolution of each map shown in the figures (at a given FSC cut-off) and the relative contour at which the map is displayed.

R: By "Surface representations of cryo-EM reconstructions," we mean cryo-EM density maps. We have now included resolution and contour level information in all relevant figure legends.

6 - Central Spike. It is unclear reading this paper if the authors see this protein or just modeled it. On line 141, they call Fig 2B, which does not show a central spike '(Fig. 2B, SFig. 1, 2J, 3, Table S1)'). Is this a mistake? In fig S2J they show the recombinant trimeric knob. But what about the coiled-coil? Was this feature ever observed? How can a reader tell if the figure shows an experimental model of pure fantasy? At times, the paper comes across as a modeling exercise.

R: Thank you. In the paragraph describing the central spike protein, we incorrectly referred to Fig. 2B instead of Fig. 3B (this has now been corrected). We did not observe the coiled-coil structure formed by three central spike domains – this region of the protein is flexible. The structure was predicted using AlphaFold2. We have now included an additional description of the central spike protein structure determination (Lines 157-161):
"The OB and β-prism domains were resolved in the reconstruction of the native phi812 baseplate (Fig. 2A). The structures of the knob and petal domains were determined by cryo-EM and single-particle reconstruction of a recombinantly produced central spike protein (Fig. 3B, Appendix Figure S1, S2, S3, Appendix Table S1). The structure of the coiled-coil domain was predicted using AlphaFold257."

We have now modified Fig. 2A to explicitly refer to the OB and β-barrel domains of the central spike protein that are resolved in the cryo-EM map. We modified Fig. 3B to define parts of the central spike protein that were predicted using AlphaFold2 and those resolved in cryo-EM

reconstruction. We modified Appendix Figure 9 to include the AlphaFold2-predicted structure of the coiled-coil domain of central spike protein colored by pLDDT score.

7 - Fig. 7. Why is the inner membrane of gram-positive labeled as OM in the diagram? Shouldn't this be a plasma membrane or an Inner Membrane (IM)? Also, what is the evidence to show that the entire tail tube penetrates the IM? Such a model is unlikely. The author should provide evidence or at least cite literature that the tail tube crossed the IM and dipped into the bacterium cytoplasm. Again, this seems odd.

R: The inner membrane was labeled CM, indicating cytoplasmic membrane. We have now changed the label to PM to stand for the plasma membrane, and we modified the figure legend accordingly (Lines 863-864):

"WTA - wall teichoic acid; LTA - lipoteichoic acid; PG - peptidoglycan; PM - plasma membrane."

8 - Table S4. X-ray data and structure quality indicators should be renamed 'X-ray data collection and refinement statistics'. Also, include the reflections in the outer shell and all relative statistics for the outer shells.

R: We have now renamed the table as requested and included the indicators for the high-resolution bin.

9 - NMR studies: why did the authors use NMR to solve the cleaver domain? It's a globular and easy-to-predict domain that AlphaFold can likely get with 99% accuracy.

R: The domain was determined previously as part of another research project.

Referee #3:

1/ The manuscript describes - to my knowledge- the first myophage infection a monoderm, as most monoderm (Firmicutes and Actinobacteria) infecting phages are siphophages and in a lesser extent podophages. This phage possess RBPs that target polysaccharides of host, and not proteinaceous receptors. Furthermore, the structure is of high quality,and, when some elements of the phages were not visible due to stistic disorder, they were predicted by AlphaFold2. Both rest and activated structure have been determined, making it possible to elaborate on the infection mechanism. The paper is well written and equisitely illustrated.
- There is no major concern.
- Minor concerns:
a) Figure 2E, assembly (not assebly)
R: Thank you. This has now been corrected.

b) In Figure 7, the RBP2 is pointing to the host cell wall (as in Figure 6C) and RBP1 is pointing to the side, which is strange for host binding). Furthermore, RBP1 is not visible in Figure6. Could you explain?
R: RBP1 is not resolved in the cryo-EM reconstruction of the contracted baseplate (Fig. 6BC). We presume the RBP1 remains attached to the baseplate after the tail contraction, but its orientation relative to the baseplate is probably variable, as it is not resolved in the reconstruction. Therefore, the orientation of RBP1 in the schematic representation shown in Fig. 7 is arbitrary. The predicted receptor binding sites are at the sides of the trimeric complexes of RBP1, therefore, it may bind to the bacterial surface in any orientation. We colored the RBP1 in Fig. 7 in stripes to indicate its arbitrary orientation. Accordingly, we modified the figure legend (Line 865):
"In panels C-E, RBP1 is colored in white-magenta stripes to indicate its variable orientation."

- Suggestions: In most recent structures (of siphophages mainly), the C-terminus of the TMP is visible. Did you see any density (helical probably) that could be assigned to the TMP? In any case, that should be commented.
R: We did not observe the density of tape measure proteins. We have now included this information (Lines 209-210):
"No density attributable to tape measure proteins was resolved in the reconstruction of the phi812 baseplate core."

Response to reviewers' comments. *Referee #2 comments are in blue italics,* **and our responses are in bold black font.**

I have invested a significant amount of time reading the revised manuscript, which I find to be an improvement over the initial submission. I commend the authors for dedicating additional time and effort to it. I believe the paper holds intellectual merit; however, in certain aspects of the structural biology workflow, it unfortunately meets a new low standard in structural virology.

Major points:

1 - Thank you for including Figures #3 and S4. It is evident from the low correlation at low resolution (<0.5) that the baseplate does not follow C3 or C6 rotational symmetry. Attempting to impose symmetry to visualize 'something' results in a map that clearly does not correlate with the data. These reconstructions should be performed without imposing any rotational symmetry. Many different conformations are clearly being averaged together, resulting in poor correlation at low resolution. The question is, how low is still acceptable in cryo-EM?
I consider these FSCs a testament to what one should avoid in single-particle analysis.
A: We collected new cryo-EM data on native phage 812 with baseplate in the pre-contraction state using Titan Krios equipped with K3 camera and recalculated the reconstructions. The resulting maps have better quality and improved FSC curves. We performed an asymmetric reconstruction of phage 812's baseplate and applied C3 symmetry only when further resolution improvement was not possible. Our results show that the baseplate in the native state obeys threefold symmetry. Accordingly, we submitted new maps to the EMDB and PDB databases and updated Fig. S3 and S4 (now Figures S1 and S7).

2 - I have examined several maps that were deposited and released, such as 9EUG, 9EUH, 9EUI, 9EUJ, and so on. Undoubtedly, these maps are of extremely low resolution, to the extent that, at best, one can segment blobs in Chimera and correlate blob shapes with AlphaFold models to identify where each factor may belong. In this respect, these maps are not significantly better than those reported in the 2016 PNAS papers. However, other parts of the tail/baseplate, where the authors were able to impose rotational symmetry, are near-atomic and sufficient to place an atomic model and apply some real-space refinement.
A: As mentioned in response to the previous point, we collected new data and carried out multiple localized reconstructions that improved the resolution and interpretability of the maps. As a result, we now provide a more comprehensive description of the baseplate.

3 - The MS table provided in the revised paper is biased by the authors' subjective ideas about what goes into the density. This is not the purpose of mass spectrometry, which should inform both the authors and the readers about the factors that are absent from the density. Selecting what belongs where based on subjective model choices for medium and low-resolution densities is a futile exercise.
A: The table provides a complete list of phage 812 proteins identified in the phage sample.

4 - I really do not intend to play the role of the police here, but I sincerely do not know where the authors could acquire 300 kV micrographs on Falcon2 and/or K2 detectors that have been obsolete for nearly a decade. As the senior author (who is an excellent microscopist) knows very well, the quality of the detector does matter, just as the data collection strategy does. It is clear that imposing C3 or C6 is not the solution for these types of baseplates. The authors should have gathered a larger dataset aimed at producing a C1 map or utilized cryo-ET to improve data quality at the single-baseplate level. The key issue with this paper is the poor quality of crucial parts of the structure, namely, the baseplate. The same virion would have yielded significantly better maps, thus requiring less guessing and modeling, if the authors had collected data on K3 or Falcon4 detectors.

A: We collected new data, using a K3 detector, to address the criticism of using micrograph recording on an obsolete detector. Using the latest, larger dataset, we calculated asymmetric reconstruction of the baseplate, which demonstrates that, in the native state, the baseplate of phage 812 conforms to C3 symmetry. Using the new data and additional sub-particle reconstructions, we indeed provide a more comprehensive description of phage 812 bseplate.

5 - The AlphAfold model fits within the 5-9A maps shown in Figure S4. These serve as tentative descriptions of macromolecules that should be clearly labeled as tentative models. Model-to-map correlations of <0.5 should not be used as a reference for any biological insights. It is unfortunate that, at a time when structural virology is striving to become atomistic, the authors relied on outdated data to extrapolate observations they could have made by simply repeating the work with current technology. As an analogy, would a genomic paper be acceptable if it utilized classical Sanger sequencing techniques? Likely not in the NGS era.

A: With improved resolution and quality of the new maps, we now provide a more complex description of the baseplate, which relies less on model fitting.

We identified a new protein at the baseplate periphery (gp122, coupler protein) present in three copies related by C3 symmetry. We identified three additional arm segment protein dimers in each baseplate arm (six in total, designated A–F), and the remaining unassigned density now aligns with the remainder of the arm-scaffold protein structure. This improves our earlier conclusion that the periphery of the baseplate is formed solely by the arm scaffold protein (see also points 7 and 8) and supports our conclusion that RBP1 and RBP2 attach to loops formed by the arm scaffold protein. This more complex structure prompted us to rename parts and domains of the arm scaffold protein. Specifically, linkers AB, BC, and Z are now named linkers 1, 2, and 3, and the connector domain is now referred to as the C-terminal domain. We resolved interactions of the linker 1 and linker 2 of the arm-scaffold protein with the arm-segment dimers, providing experimental evidence that scaffold linkers form a β-strand between the C-terminal β-strands of two arm-segment proteins. This evidence supports our hypothesis and that of others (Guerrero-Ferreira et al., 2019) that the arm segment protein dimers reinforce the arm scaffold linkers (see also point 12). We resolved the bulge domain of the arm-scaffold protein, confirming its correct placement in the previous low-resolution maps of the pre-contraction baseplate. We resolved the attachment sites of inner and outer tripod protein trimers to the docking loops 1 and 2 of the arm scaffold protein, respectively. This provides experimental evidence that a) the attachment mode remains unchanged before and after contraction (see also point 14), and b) the docking loops 1

and 2 of the arm scaffold protein form a similar triangular structure with the residue pattern predicted earlier only from a sequence alignment. We partially resolved the elbow domain of the arm scaffold protein, experimentally refining its placement at the periphery of the arm.

6 - Figure 7E. There is no evidence that the baseplate penetrated the cytoplasmic membrane. This suggests that it would puncture a large hole in the host, likely resulting in membrane depolarization and cell death. From a biological perspective, this model is implausible. Cryo-ET studies with T4 found that the baseplate stops at the peptidoglycan. This is clearly different in Gram-positive bacteria, but the idea that a >400 Å structure penetrates through a lipid bilayer defies reality.

A: Indeed, there is no evidence that phage 812 baseplate penetrates the cytoplasmic membrane, as shown in Fig. S16, and previously by Nováček et al. 2016, the baseplate remains attached to the surface of the bacterial cell wall. However, our results provide evidence that the tip of phage 812 tail tube reaches the cytoplasm (this has been establisghed in previous publication by Nováček et al, 2016): Upon contraction the tail sheath pf 812 shortens from 200 to 96 nm whereas the cell wall of S. aureus is 50-70nm thick, therefore, the tip of the tail tube reaches the cytoplasm.

Overall, I find phage 812 to be very interesting: there is a pressing need for a better understanding of Gram-positive phages. This paper builds upon the 2016 PNAS paper and improves it. The authors have progressed from very low resolution (e.g., segmented volumes) to a medium resolution that is sufficient for docking AlphaFold models. The paper is quite comprehensive; it presents a substantial amount of data, with some being high quality and a portion being low quality. Overall, this work signifies progress in the field but does not constitute a comprehensive molecular atlas of the first Gram-positive phage.

A: We hope that with the new data and additional reconstruction, our work provides an adequate addition to our understanding of phages infecting Gram-positive bacteria.

Dr. Pavel Plevka
CEITEC, Masaryk University
Structural virology
Kamenice 5/E35
Brno 62500
Czech Republic

27th May 2025

Re: EMBOJ-2025-120378R
Conformational changes of baseplate linked to tail contraction of S. aureus phage phi812

Dear Dr. Plevka,

Thank you again for submitting your revised manuscript on phage phi812 baseplate structural dynamics for our consideration. I sent it back to the original reviewer 2, who had initially raised the most serious concerns regarding the structural analysis, resolution, and modeling aspects of the work, to see whether they would be satisfied with the responses and revisions. I am afraid to say that, after having had the chance to assess the underlying raw data in more detail, our expert referee was not convinced that the analyzed data are of sufficient quality to warrant the structural modeling in the manuscript. In light of these major concerns, we unfortunately had to conclude that we cannot offer publication of this study in The EMBO Journal after all.

As you will see from the comments below, although the referee appreciates that the study addresses an important subject, presents a lot of data, and contains some valuable information, s/he maintains that many of the experimental maps are of too low resolution to allow definitive novel conclusions. Furthermore, the reviewer emphasizes that structural modeling should be limited to cases in which current state-of-the-art methodologies are not able to yield higher definitions, which does not appear to be the case here. I will not repeat all their individual points of criticism in detail here, but hope you understand that analyses at the highest technical level and up to current methodological standards remain a pre-requisite for publication in The EMBO Journal.

While we cannot pursue this manuscript further, we encourage you to transfer your study to our not-for-profit open-access sister journal, Life Science Alliance (LSA). We shared your manuscript and the accompanying reviews with LSA Executive Editor, Tim Fessenden, who is interested in these findings. He is pleased to offer publication of this manuscript at LSA pending the following revisions:
- Revise claims in the abstract, results, and discussion in light of the overall low correlation values shown in Figure S4, which Reviewer 2 stipulates would have to be termed "tentative". This includes claims on C3 or C6 symmetry, which should be recast as speculative at this resolution.
- Clearly state instrumentation used, and limits on resolution, in light of Reviewer 2 point 4.
- Alter the model shown in Fig 7 in light of Reviewer 2 point 6.

We understand that such a revision might need to be re-reviewed, in which case Dr. Fessenden will walk the Reviewers through our transfer process. We encourage you to use the link below to transfer your manuscript to LSA. You do not need to revise the manuscript before transferring it to LSA. Once you transfer, Dr. Fessenden will email you an invitation to revise and resubmit, listing the same revision requests as mentioned above. Please feel free to reach out at t.fessenden@life-science-alliance.org if you have any questions about the LSA journal, the transfer process, or the revisions requested.

I am sorry that the referee feedback did not allow me to be more positive for The EMBO Journal, despite the significant amount of additional work and effort you have invested here since the original submission, but hope you will still find this transfer option valuable.

Yours sincerely,

Hartmut Vodermaier

Hartmut Vodermaier, PhD
Senior Editor, The EMBO Journal
h.vodermaier@embojournal.org

****

Referee #2:

I have invested a significant amount of time reading the revised manuscript, which I find to be an improvement over the initial submission. I commend the authors for dedicating additional time and effort to it. I believe the paper holds intellectual merit; however, in certain aspects of the structural biology workflow, it unfortunately meets a new low standard in structural virology.

Major points:

1 - Thank you for including Figures #3 and S4. It is evident from the low correlation at low resolution (<0.5) that the baseplate does not follow C3 or C6 rotational symmetry. Attempting to impose symmetry to visualize 'something' results in a map that clearly does not correlate with the data. These reconstructions should be performed without imposing any rotational symmetry. Many different conformations are clearly being averaged together, resulting in poor correlation at low resolution. The question is, how low is still acceptable in cryo-EM?
I consider these FSCs a testament to what one should avoid in single-particle analysis.

2 - I have examined several maps that were deposited and released, such as 9EUG, 9EUH, 9EUI, 9EUJ, and so on. Undoubtedly, these maps are of extremely low resolution, to the extent that, at best, one can segment blobs in Chimera and correlate blob shapes with AlphaFold models to identify where each factor may belong. In this respect, these maps are not significantly better than those reported in the 2016 PNAS papers. However, other parts of the tail/baseplate, where the authors were able to impose rotational symmetry, are near-atomic and sufficient to place an atomic model and apply some real-space refinement.

3 - The MS table provided in the revised paper is biased by the authors' subjective ideas about what goes into the density. This is not the purpose of mass spectrometry, which should inform both the authors and the readers about the factors that are absent from the density. Selecting what belongs where based on subjective model choices for medium and low-resolution densities is a futile exercise.

4 - I really do not intend to play the role of the police here, but I sincerely do not know where the authors could acquire 300 kV micrographs on Falcon2 and/or K2 detectors that have been obsolete for nearly a decade. As the senior author (who is an excellent microscopist) knows very well, the quality of the detector does matter, just as the data collection strategy does. It is clear that imposing C3 or C6 is not the solution for these types of baseplates. The authors should have gathered a larger dataset aimed at producing a C1 map or utilized cryo-ET to improve data quality at the single-baseplate level. The key issue with this paper is the poor quality of crucial parts of the structure, namely, the baseplate. The same virion would have yielded significantly better maps, thus requiring less guessing and modeling, if the authors had collected data on K3 or Falcon4 detectors.

5 - The AlphAfold model fits within the 5-9A maps shown in Figure S4. These serve as tentative descriptions of macromolecules that should be clearly labeled as tentative models. Model-to-map correlations of <0.5 should not be used as a reference for any biological insights. It is unfortunate that, at a time when structural virology is striving to become atomistic, the authors relied on outdated data to extrapolate observations they could have made by simply repeating the work with current technology. As an analogy, would a genomic paper be acceptable if it utilized classical Sanger sequencing techniques? Likely not in the NGS era.

6 - Figure 7E. There is no evidence that the baseplate penetrated the cytoplasmic membrane. This suggests that it would puncture a large hole in the host, likely resulting in membrane depolarization and cell death. From a biological perspective, this model is implausible. Cryo-ET studies with T4 found that the baseplate stops at the peptidoglycan. This is clearly different in Gram-positive bacteria, but the idea that a >400 Å structure penetrates through a lipid bilayer defies reality.

Overall, I find phage 812 to be very interesting: there is a pressing need for a better understanding of Gram-positive phages. This paper builds upon the 2016 PNAS paper and improves it. The authors have progressed from very low resolution (e.g., segmented volumes) to a medium resolution that is sufficient for docking AlphaFold models. The paper is quite comprehensive; it presents a substantial amount of data, with some being high quality and a portion being low quality. Overall, this work signifies progress in the field but does not constitute a comprehensive molecular atlas of the first Gram-positive phage.

*** As a service to authors, The EMBO Journal offers the possibility to directly transfer declined manuscripts to another EMBO Press title (EMBO Reports, EMBO Molecular Medicine, Molecular Systems Biology) or to the open access journal Life Science Alliance launched in partnership between EMBO Press, Rockefeller University Press and Cold Spring Harbor Laboratory Press. The full manuscript (including reviewer comments, where applicable and if chosen) will be automatically forwarded to the receiving journal, to allow for fast handling and a prompt decision on your manuscript. For more details of this service, and to transfer your manuscript to another EMBO title please follow this link:

This document summarizes new data and insights since the last reviewed version of the manuscript: "Conformational changes of baseplate regulating tail contraction of Staphylococcus phage 812" by Bíňovský et al. The summary is divided into two sections: pre-contraction and post-contraction baseplate. Due to the new data, all main text figures and most supplementary figures were modified.

Pre-contraction baseplate

New data

- We collected new cryo-EM data on phage 812 with the baseplate in the pre-contraction state.
- Data were recorded using a K3 camera, resulting in higher-quality reconstructions and improved FSC curves.
- We first performed an asymmetric reconstruction (C1) of the baseplate and applied the C3 symmetry only when further improvement in resolution was not possible. We demonstrate that the baseplate of phage 812 has threefold symmetry.
- We carried out multiple localized reconstructions that improved interpretability, model building, and fitting.

New insights that refine or substantiate our conclusions:

1. An asymmetric reconstruction using an initial model low-pass-filtered to 60 Å naturally converged to three-fold symmetry, providing experimental evidence that the C3 symmetry of the pre-contraction baseplate is intrinsic and not an artifact imposed by us.
2. We identified a new protein at the baseplate periphery (gp122, coupler protein) present in three copies related by C3 symmetry.
3. We identified three additional arm segment protein dimers in each baseplate arm (six in total, designated A–F), and the remaining unassigned density now aligns with the remainder of the arm-scaffold protein structure. This improves our earlier conclusion that the periphery of the baseplate is formed solely by the arm scaffold protein (see also points 7 and 8) and supports our conclusion that RBP1 and RBP2 attach to loops formed by the arm scaffold protein. This more complex structure prompted us to rename parts and domains of the arm scaffold protein. Specifically, linkers AB, BC, and Z are now named linkers 1, 2, and 3, and the connector domain is now referred to as the C-terminal domain.
4. We resolved interactions of the linker 1 and linker 2 of the arm-scaffold protein with the arm-segment dimers, providing experimental evidence that scaffold linkers form a β-strand between the C-terminal β-strands of two arm-segment proteins. This evidence supports our hypothesis and that of others (Guerrero-Ferreira et al., 2019) that the arm segment protein dimers reinforce the arm scaffold linkers (see also point 12).
5. We resolved the bulge domain of the arm-scaffold protein, confirming its correct placement in the previous low-resolution maps of the pre-contraction baseplate.
6. We resolved the attachment sites of inner and outer tripod protein trimers to the docking loops 1 and 2 of the arm scaffold protein, respectively. This provides experimental evidence

that a) the attachment mode remains unchanged before and after contraction (see also point 14), and b) the docking loops 1 and 2 of the arm scaffold protein form a similar triangular structure with the residue pattern predicted earlier only from a sequence alignment.

7. We partially resolved the elbow domain of the arm scaffold protein, experimentally refining its placement at the periphery of the arm.
8. The unassigned densities at the end of baseplate arms now match the C-terminal domain of the arm-scaffold protein in shape and volume.
9. We resolved parts of the tripod trimers, providing experimental evidence of their presence, correct placement, and intra-molecular conformation in the pre-contraction baseplate.
10. We resolved parts of RBP1 and RBP2 trimers, providing experimental evidence of their presence and correct placement in the pre-contraction baseplate.

Post-contraction baseplate
New data:

- We reprocessed the cryo-EM dataset of phage 812 with the baseplate in the post-contraction state, starting with motion-corrected micrographs and picked particle coordinates. We first performed an asymmetric reconstruction and applied the C6 symmetry only when further improvement in resolution was not possible.
- We conducted two additional localized reconstructions of the baseplate arm, which improved interpretability, model building, and fitting.

New insights that refine or substantiate our conclusions:

11. An asymmetric reconstruction using a 60 Å low-pass-filtered starting model naturally converged to six-fold symmetry, providing experimental evidence that the C6 symmetry of the post-contraction baseplate is intrinsic and not an artifact imposed by us.
12. Similar to point 4 above, we resolved interactions of the linker 1 and linker 2 of the arm-scaffold protein with the arm-segment dimers. This provides experimental evidence that the interactions remain unchanged in the post-contraction baseplate.
13. Similar to point 5 above, we resolved the bulge domain of the arm-scaffold protein, confirming its correct placement in the previous low-resolution maps of the post-contraction baseplate.
14. We resolved the attachment of the outer tripod protein trimer to the docking loop 2 of the arm scaffold protein. The substantiated conclusions are explained in point 6 above.
15. We resolved parts of the outer tripod trimer, providing experimental evidence of its correct placement and the same conformation as that of the inner tripod in the post-contraction baseplate.

As a consequence of the new data and insights described above, the text, main and supplementary figures, and supplementary tables were modified.

Response to reviewers' comments. *Referee #2 comments are in blue italics,* **and our responses are in bold black font.**

I have invested a significant amount of time reading the revised manuscript, which I find to be an improvement over the initial submission. I commend the authors for dedicating additional time and effort to it. I believe the paper holds intellectual merit; however, in certain aspects of the structural biology workflow, it unfortunately meets a new low standard in structural virology.

Major points:

1 - Thank you for including Figures #3 and S4. It is evident from the low correlation at low resolution (<0.5) that the baseplate does not follow C3 or C6 rotational symmetry. Attempting to impose symmetry to visualize 'something' results in a map that clearly does not correlate with the data. These reconstructions should be performed without imposing any rotational symmetry. Many different conformations are clearly being averaged together, resulting in poor correlation at low resolution. The question is, how low is still acceptable in cryo-EM?
I consider these FSCs a testament to what one should avoid in single-particle analysis.
A: We collected new cryo-EM data on native phage 812 with baseplate in the pre-contraction state using Titan Krios equipped with K3 camera and recalculated the reconstructions. The resulting maps have better quality and improved FSC curves. We performed an asymmetric reconstruction of phage 812's baseplate and applied C3 symmetry only when further resolution improvement was not possible. Our results show that the baseplate in its native state exhibits threefold symmetry. Accordingly, we submitted new maps to the EMDB and PDB databases and updated Fig. S3 and S4 (now Figures S1 and S7).

2 - I have examined several maps that were deposited and released, such as 9EUG, 9EUH, 9EUI, 9EUJ, and so on. Undoubtedly, these maps are of extremely low resolution, to the extent that, at best, one can segment blobs in Chimera and correlate blob shapes with AlphaFold models to identify where each factor may belong. In this respect, these maps are not significantly better than those reported in the 2016 PNAS papers. However, other parts of the tail/baseplate, where the authors were able to impose rotational symmetry, are near-atomic and sufficient to place an atomic model and apply some real-space refinement.
A: As mentioned in response to the previous point, we collected new data and carried out multiple localized reconstructions that improved the resolution and interpretability of the maps. As a result, we now provide a more comprehensive description of the baseplate.

3 - The MS table provided in the revised paper is biased by the authors' subjective ideas about what goes into the density. This is not the purpose of mass spectrometry, which should inform both the authors and the readers about the factors that are absent from the density. Selecting what belongs where based on subjective model choices for medium and low-resolution densities is a futile exercise.
A: The table provides a complete list of phage 812 proteins identified in the phage sample.

4 - I really do not intend to play the role of the police here, but I sincerely do not know where the authors could acquire 300 kV micrographs on Falcon2 and/or K2 detectors that have been obsolete for nearly a decade. As the senior author (who is an excellent microscopist) knows very well, the quality of the detector does matter, just as the data collection strategy does. It is clear that imposing C3 or C6 is not the solution for these types of baseplates. The authors should have gathered a larger dataset aimed at producing a C1 map or utilized cryo-ET to improve data quality at the single-baseplate level. The key issue with this paper is the poor quality of crucial parts of the structure, namely, the baseplate. The same virion would have yielded significantly better maps, thus requiring less guessing and modeling, if the authors had collected data on K3 or Falcon4 detectors.

A: We collected new data, using a K3 detector, to address the criticism of using micrograph recording on an obsolete detector. Using the latest, larger dataset, we calculated the asymmetric reconstruction of the baseplate, demonstrating that, in the native state, the baseplate of phage 812 conforms to C3 symmetry. Using the new data and additional sub-particle reconstructions, we indeed provide a more comprehensive description of phage 812 baseplate.

5 - The AlphAfold model fits within the 5-9A maps shown in Figure S4. These serve as tentative descriptions of macromolecules that should be clearly labeled as tentative models. Model-to-map correlations of <0.5 should not be used as a reference for any biological insights. It is unfortunate that, at a time when structural virology is striving to become atomistic, the authors relied on outdated data to extrapolate observations they could have made by simply repeating the work with current technology. As an analogy, would a genomic paper be acceptable if it utilized classical Sanger sequencing techniques? Likely not in the NGS era.

A: With improved resolution and quality of the new maps, we now provide a more complex description of the baseplate, which relies less on model fitting.

We identified a new protein at the baseplate periphery (gp122, coupler protein). We identified three additional arm segment protein dimers in each baseplate arm (six in total, designated A–F), and the remaining unassigned density now aligns with the remainder of the arm-scaffold protein structure. This improves our earlier conclusion that the periphery of the baseplate is formed solely by the arm scaffold protein (please see also our responses to points 7 and 8 below) and supports our conclusion that RBP1 and RBP2 attach to loops formed by the arm scaffold protein. The updated structure prompted us to rename parts and domains of the arm scaffold protein. Specifically, linkers AB, BC, and Z are now named linkers 1, 2, and 3, and the connector domain is now referred to as the C-terminal domain. We resolved interactions of the linker 1 and linker 2 of the arm-scaffold protein with the arm-segment dimers, providing experimental evidence that scaffold linkers form a β-strand between the C-terminal β-strands of two arm-segment proteins. This evidence supports our hypothesis and that of others (Guerrero-Ferreira et al., 2019) that the arm segment protein dimers reinforce the linkers of the arm scaffold protein (see also our response to point 12). We resolved the structure of the bulge domain of the arm-scaffold protein, confirming its correct placement in the previous low-resolution maps of the pre-contraction baseplate. We resolved the attachment sites of inner and outer tripod protein trimers to the docking loops 1 and 2 of the arm scaffold protein, respectively. This provides experimental evidence that a) the attachment mode remains unchanged before and after contraction (see also our response to point 14), and

b) the docking loops 1 and 2 of the arm scaffold protein form a similar three-pointed star-like structure with the residue pattern predicted earlier only from a sequence alignment. We partially resolved the elbow domain of the arm scaffold protein, experimentally refining its placement at the periphery of the baseplate arm.

6 - Figure 7E. There is no evidence that the baseplate penetrated the cytoplasmic membrane. This suggests that it would puncture a large hole in the host, likely resulting in membrane depolarization and cell death. From a biological perspective, this model is implausible. Cryo-ET studies with T4 found that the baseplate stops at the peptidoglycan. This is clearly different in Gram-positive bacteria, but the idea that a >400 Å structure penetrates through a lipid bilayer defies reality.

A: Indeed, there is no evidence that phage 812 baseplate penetrates the cytoplasmic membrane - as shown in Fig. S16, and previously by Nováček et al. 2016, the baseplate of phi812 remains attached at the surface of the bacterial cell wall. However, our results provide evidence that the tip of phage 812 tail tube reaches the cytoplasm (Nováček et al, 2016): Upon contraction, the tail sheath of 812 shortens from 200 to 96 nm, whereas the cell wall of S. aureus is 50-70nm thick; therefore, the tip of the tail tube reaches the cytoplasm. Please note that the tail of phage T4 is significantly shorter than that of phage 812, and, indeed, the tip of the T4 tail tube does not reach into the cytoplasm; however, it penetrates the outer bacterial membrane.

Overall, I find phage 812 to be very interesting: there is a pressing need for a better understanding of Gram-positive phages. This paper builds upon the 2016 PNAS paper and improves it. The authors have progressed from very low resolution (e.g., segmented volumes) to a medium resolution that is sufficient for docking AlphaFold models. The paper is quite comprehensive; it presents a substantial amount of data, with some being high quality and a portion being low quality. Overall, this work signifies progress in the field but does not constitute a comprehensive molecular atlas of the first Gram-positive phage.

A: We hope that with the new data and additional reconstructions, our work provides an adequate addition to our understanding of phages infecting Gram-positive bacteria.

1st Revision - Editorial Decision 14th Apr 2026

Dr. Pavel Plevka
CEITEC, Masaryk University
Structural virology
Kamenice 5/E35
Brno 62500
Czech Republic

14th Apr 2026

Re: EMBOJ-2025-120378R1-Q
Conformational changes of baseplate regulating tail contraction of Staphylococcus phage 812

Dear Dr. Plevka,

Thank you for resubmitting a new version of your manuscript on phage 812 baseplate structures to The EMBO Journal. I sent it once more to the original referee 2, and I am happy to say that they found the paper substantially improved and are now supportive of its publication, as soon as a few minor presentational changes (please see their comments below) have been incorporated. In addition, there are a few editorial issues that I would ask you to take care of at this stage:

- Co-author Maryna Zlatohurska is listed on the article front page but needs to also be entered in our submission system.

- Similarly, all funding information listed in the manuscript needs to also be properly entered into our submission system (not just using the 'comments' box).

- Please complete our author checklist (see download link below), and upload it with the final manuscript.

- Please also once more complete an updated Source Data checklist (downloadable at https://www.embopress.org/page/journal/14693178/authorguide), detailing if figures are based on deposited source data, or on source data uploaded with the submission.

- Please upload main text (including figure legends) and main figures (without legend text) separately as individual (production-quality) files.

- Please adjust the order of the manuscript sections, and also make sure to use the correct section headers: Title page with complete author information, Abstract, Introduction, Results, Discussion (or Results and Discussion), Methods, Data Availability, Acknowledgements, Disclosure and Competing Interests Statement, References, Main Figure Legends, Tables, Expanded Figure Legends.

- Please carefully go through the reference list, which contains several journal article entries lacking full citation information such as volume or page/eLocator numbers.

- All Materials and Methods need to be described in the main text using our 'Structured Methods' format. The Methods section should include a (separately uploaded) Reagents and Tools Table (template downloadable at https://www.embopress.org/page/journal/14693178/authorguide) listing key reagents, experimental models, software and relevant equipment, and including their sources and relevant identifiers; followed by a Methods and Protocols section describing the methods (ideally using a step-by-step protocol format to facilitate adoption of the methodologies across labs)

- Please include the Code Availability as part of the Data Availability section, rather than a separate section. Also, please make sure to spell out hyperlinks to EMDB and PDB at the beginning of the section, rather than including embedded hyperlinks.

- Please check that each figure panel is referenced at least once in the text (some callouts are currently missing), and that all callouts appear sequentially - if necessary, you may first call-out the complete figure (e.g., Fig 3A-E) before referencing individual panels. Also double-check the Appendix item callouts, e.g. references to Appendix Figure 5 and Appendix Table 1 should be corrected to Appendix Figure S5 and Appendix Table S1.

- In the Appendix PDF, please expand the table of contents to list each item (with page number) separately, instead of just referring to 'Appendix Figures' or 'Appendix Tables' collectively.

- Finally, please provide suggestions for a short 'blurb' text prefacing and summing up the conceptual aspect of the study in two sentences (max. 250 characters), followed by 3-5 one-sentence 'bullet points' with brief factual statements of key results of the

paper; they will form the basis of an editor-written 'Synopsis' accompanying the online version of the article. Please also upload a synopsis image, which can be used as a "visual title" for the synopsis section of your paper. The image should be in JPG format, and please make sure that it remains in the modest dimensions of (exactly) 550 pixels wide and 300 pixels high.

I am returning the manuscript to you for a final round of minor revision, to allow you to make these remaining modifications and upload the revised files. Once we will have received them, we should be ready to swiftly proceed with formal acceptance and production of the manuscript.

With kind regards,

Hartmut Vodermaier

Hartmut Vodermaier, PhD
Senior Editor, The EMBO Journal
h.vodermaier@embojournal.org

*** PLEASE NOTE: All revised manuscript are subject to initial checks for completeness and adherence to our formatting guidelines. Revisions may be returned to the authors and delayed in their editorial re-evaluation if they fail to comply to the following requirements. As a first step please read our guidelines for revised submissions:
https://link.springer.com/journal/44318/submission-guidelines#cms-Revised-submissions

1) Every manuscript requires a Data Availability section (even if only stating that no deposited datasets are included). Primary datasets or computer code produced in the current study have to be deposited in appropriate public repositories prior to resubmission, and reviewer access details provided in case that public access is not yet allowed.

2) Each figure legend must specify
- size of the scale bars that are mandatory for all micrograph panels
- the statistical test used to generate error bars and P-values
- the type error bars (e.g., S.E.M., S.D.)
- the number (n) and nature (biological or technical replicate) of independent experiments underlying each data point
- Figures may not include error bars for experiments with n<3; scatter plots showing individual data points should be used instead.

3) Revised manuscript text (including main tables, and figure legends for main and EV figures) has to be submitted as editable text file (e.g., .docx format). We encourage highlighting of changes (e.g., via text color) for the referees' reference.

4) Each main and each Expanded View (EV) figure should be uploaded as individual production-quality files (preferably in .eps, .tif, .jpg formats). For suggestions on figure preparation/layout, please refer to our Figure Preparation Guidelines:
https://media.springernature.com/original/springer-cms/rest/v1/content/27825798/data/v1

5) Point-by-point response letters should include the original referee comments in full together with your detailed responses to them (and to specific editor requests if applicable), and also be uploaded as editable (e.g., .docx) text files.

6) Please complete our Author Checklist, and make sure that information entered into the checklist is also reflected in the manuscript; the checklist will be available to readers as part of the Review Process File.

7) All authors listed as (co-)corresponding need to deposit, in their respective author profiles in our submission system, a unique ORCiD identifier linked to their name. Please see our Guide to Authors for detailed instructions.

8) Please note that supplementary information at EMBO Press has been superseded by the 'Expanded View' for inclusion of additional figures, tables, movies or datasets; with up to five EV Figures being typeset and directly accessible in the HTML version of the article.

9) To facilitate reproducibilty and cross-laboratory adoption of methodologies, please structure the Materials & Methods section as outlined in our guide to authors, including a completed Reagents and Tools Table.

10) Digital image enhancement is acceptable practice, as long as it accurately represents the original data and conforms to community standards. If a figure has been subjected to significant electronic manipulation, this must be clearly noted in the figure legend and/or the 'Materials and Methods' section. The editors reserve the right to request original versions of figures and the original images that were used to assemble the figure. Finally, we generally encourage uploading of numerical as well as gel/blot image source data.

At EMBO Press we ask authors to provide source data for the main manuscript figures. You will receive a separate email with instructions for providing source data with your revised manuscript, including how to upload and organize the files.

In the interest of ensuring the conceptual advance provided by the work, we recommend submitting a revision within 3 months (13th Jul 2026). Please discuss the revision progress ahead of this time with the editor if you require more time to complete the revisions. Use the link below to submit your revision:

Link Not Available

Referee #2:

The manuscript has been substantially improved and now presents a more accurate and refined analysis. The inclusion of 300 kV data is an important addition, leading to improved map quality and more reliable models. Although the resolution of the baseplate arms in the pre-contraction state remains relatively low (4-7 Å), the use of AlphaFold3 models enables a reasonably precise structural interpretation. Overall, the study represents a meaningful conceptual advance and makes a significant contribution to the field of structural virology.

Minor points:

1 - The nomenclature of the baseplate remains somewhat complex and repetitive (e.g., core, arms, petals, wedges). The authors should consider simplifying the terminology, as the current naming scheme risks making the architecture appear more complicated than it is. Alternatively, these elements should be clearly labeled, especially in Fig. 7

2 - The terminology should be standardized or clarified for "contracted baseplate" versus "contracted triplex," as the distinction between these terms is currently unclear.

3 - Appendix Fig. S1. The color scale should be consistent across all reconstructions. If the color coding varies with resolution, it may give the impression that all maps have similar resolution. The authors should define a fixed range (e.g., 8-3 Å) and apply it uniformly.

Reviewer's comments are in blue italics, **and our responses are in black bold font.**

Referee #2:

The manuscript has been substantially improved and now presents a more accurate and refined analysis. The inclusion of 300 kV data is an important addition, leading to improved map quality and more reliable models. Although the resolution of the baseplate arms in the pre-contraction state remains relatively low (4-7 Å), the use of AlphaFold3 models enables a reasonably precise structural interpretation. Overall, the study represents a meaningful conceptual advance and makes a significant contribution to the field of structural virology.

Minor points:

1 - The nomenclature of the baseplate remains somewhat complex and repetitive (e.g., core, arms, petals, wedges). The authors should consider simplifying the terminology, as the current naming scheme risks making the architecture appear more complicated than it is. Alternatively, these elements should be clearly labeled, especially in Fig. 7
A: The nomenclature of baseplate components is based on previous publications; however, we agree that it is complex. We have now included graphical representations of baseplate components in Fig. 1. In addition, Fig. 7 includes a color legend identifying baseplate components.

2 - The terminology should be standardized or clarified for "contracted baseplate" versus "contracted triplex," as the distinction between these terms is currently unclear.
A: We checked the manuscript, and it does not contain the expression "contracted triplex".

3 - Appendix Fig. S1. The color scale should be consistent across all reconstructions. If the color coding varies with resolution, it may give the impression that all maps have similar resolution. The authors should define a fixed range (e.g., 8-3 Å) and apply it uniformly.
A: We did not modify the color scales of the individual figures according to the reviewer's suggestion. Because of major differences in the resolutions of the individual maps and, therefore, the need for a rather broad range common to all the figures, each re-colored map would show only one color. This would allow comparison of overall resolutions among the maps, but, in our opinion, this is not the purpose of this type of display and is not a standard used in the field. The purpose of resolution-color-coded maps is to show which parts of each map are well resolved and which are less well resolved. That is why they have individually adjusted scales.

EMBO Press Author Checklist

Corresponding Author Name: Pavel Plevka	
Journal Submitted to: The EMBO Journal	
Manuscript Number: EMBOJ-2025-120378R1-Q	

USEFUL LINKS FOR COMPLETING THIS FORM
The EMBO Journal - Author Guidelines
EMBO Reports - Author Guidelines
Molecular Systems Biology - Author Guidelines
EMBO Molecular Medicine - Author Guidelines

Reporting Checklist for Life Science Articles (updated January
This checklist is adapted from Materials Design Analysis Reporting (MDAR) Checklist for Authors. MDAR establishes a minimum set of requirements in transparent reporting in the life sciences (see Statement of Task: 10.31222/osf.io/9sm4x). Please follow the journal's guidelines in preparing your
Please note that a copy of this checklist will be published alongside your article.

Abridged guidelines for figures
1. Data
The data shown in figures should satisfy the following conditions:
- the data were obtained and processed according to the field's best practice and are presented to reflect the results of the experiments in an accurate and unbiased manner.
- ideally, figure panels should include only measurements that are directly comparable to each other and obtained with the same assay.
- plots include clearly labeled error bars for independent experiments and sample sizes. Unless justified, error bars should not be shown for technical
- if n<5, the individual data points from each experiment should be plotted. Any statistical test employed should be justified.
- Source Data should be included to report the data underlying figures according to the guidelines set out in the authorship guidelines on Data

2. Captions
Each figure caption should contain the following information, for each panel where they are relevant:
- a specification of the experimental system investigated (eg cell line, species name).
- the assay(s) and method(s) used to carry out the reported observations and measurements.
- an explicit mention of the biological and chemical entity(ies) that are being measured.
- an explicit mention of the biological and chemical entity(ies) that are altered/varied/perturbed in a controlled manner.
- the exact sample size (n) for each experimental group/condition, given as a number, not a range;
- a description of the sample collection allowing the reader to understand whether the samples represent technical or biological replicates (including how many animals, litters, cultures, etc.).
- a statement of how many times the experiment shown was independently replicated in the laboratory.
- definitions of statistical methods and measures:
  - common tests, such as t-test (please specify whether paired vs. unpaired), simple χ2 tests, Wilcoxon and Mann-Whitney tests, can be unambiguously identified by name only, but more complex techniques should be described in the methods section;
  - are tests one-sided or two-sided?
  - are there adjustments for multiple comparisons?
  - exact statistical test results, e.g., P values = x but not P values < x;
  - definition of 'center values' as median or average;
  - definition of error bars as s.d. or s.e.m.

Please complete ALL of the questions below.
Select "Not Applicable" only when the requested information is not relevant for your study.

Materials

Newly Created Materials	Information included in the manuscript?	In which section is the information available? (Reagents and Tools Table, Materials and Methods, Figures, Data Availability Section)
New materials and reagents need to be available; do any restrictions apply?	Not Applicable	

Antibodies	Information included in the manuscript?	In which section is the information available? (Reagents and Tools Table, Materials and Methods, Figures, Data Availability Section)
For **antibodies** provide the following information:<br>- Commercial antibodies: RRID (if possible) or supplier name, catalogue number and or/clone number<br>- Non-commercial: RRID or citation	Not Applicable	

DNA and RNA sequences	Information included in the manuscript?	In which section is the information available? (Reagents and Tools Table, Materials and Methods, Figures, Data Availability Section)
**Short novel DNA or RNA including primers, probes**: provide the sequences.	Yes	Materials and Methods

Cell materials	Information included in the manuscript?	In which section is the information available? (Reagents and Tools Table, Materials and Methods, Figures, Data Availability Section)
**Cell lines:** Provide species information, strain. Provide accession number in repository **OR** supplier name, catalog number, clone number, and/**OR** RRID.	Not Applicable	
**Primary cultures:** Provide species, strain, sex of origin, genetic modification status.	Not Applicable	
Report if the cell lines were recently **authenticated** (e.g., by STR profiling) and tested for mycoplasma contamination.	Not Applicable	

Experimental animals	Information included in the manuscript?	In which section is the information available? (Reagents and Tools Table, Materials and Methods, Figures, Data Availability Section)
**Laboratory animals or Model organisms:** Provide species, strain, sex, age, genetic modification status. Provide accession number in repository **OR** supplier name, catalog number, clone number, **OR** RRID.	Not Applicable	
**Animal observed in or captured from the field:** Provide species, sex, and age where possible**.**	Not Applicable	
Please detail **housing and husbandry conditions**.	Not Applicable	

Plants and microbes	Information included in the manuscript?	In which section is the information available? (Reagents and Tools Table, Materials and Methods, Figures, Data Availability Section)
**Plants:** provide species and strain, ecotype and cultivar where relevant, unique accession number if available, and source (including location for collected wild specimens).	Not Applicable	
**Microbes:** provide species and strain, unique accession number if available, and source.	Yes	Materials and Methods, Reagents and Tools Table

Human research participants	Information included in the manuscript?	In which section is the information available? (Reagents and Tools Table, Materials and Methods, Figures, Data Availability Section)
If collected and within the bounds of privacy constraints report on age, sex and gender or ethnicity for all study participants.	Not Applicable	

Core facilities	Information included in the manuscript?	In which section is the information available? (Reagents and Tools Table, Materials and Methods, Figures, Data Availability Section)
If your work benefited from core facilities, was their service mentioned in the acknowledgments section?	Yes	Acknowledgments

Design

Study protocol	Information included in the manuscript?	In which section is the information available? (Reagents and Tools Table, Materials and Methods, Figures, Data Availability Section)
If study protocol has been **pre-registered, provide DOI in the manuscript**. For clinical trials, provide the trial registration number **OR** cite DOI.	Not Applicable	
Report the **clinical trial registration number** (at ClinicalTrials.gov or equivalent), where applicable.	Not Applicable	

Laboratory protocol	Information included in the manuscript?	In which section is the information available? (Reagents and Tools Table, Materials and Methods, Figures, Data Availability Section)
Provide DOI OR other citation details if **external detailed step-by-step protocols** are available.	Not Applicable	

Experimental study design and statistics	Information included in the manuscript?	In which section is the information available? (Reagents and Tools Table, Materials and Methods, Figures, Data Availability Section)
Include a statement about **sample size** estimate even if no statistical methods were used.	Not Applicable	
Were any steps taken to minimize the effects of subjective bias when allocating animals/samples to treatment (e.g. **randomization procedure**)? If yes, have they been described?	Not Applicable	
Include a statement about **blinding** even if no blinding was done.	Not Applicable	
Describe **inclusion/exclusion criteria** if samples or animals were excluded from the analysis. Were the criteria pre-established?<br><br>If sample or data points were omitted from analysis, report if this was due to attrition or intentional exclusion and provide justification.	Not Applicable	
For every figure, are **statistical tests** justified as appropriate? Do the data meet the assumptions of the tests (e.g., normal distribution)? Describe any methods used to assess it. Is there an estimate of variation within each group of data? **I**s the variance similar between the groups that are being statistically compared?	Not Applicable	

Sample definition and in-laboratory replication	Information included in the manuscript?	In which section is the information available? (Reagents and Tools Table, Materials and Methods, Figures, Data Availability Section)
In the figure legends: state number of times the experiment was **replicated** in laboratory.	Not Applicable	
In the figure legends: define whether data describe **technical or biological replicates**.	Not Applicable	

Ethics

Ethics	Information included in the manuscript?	In which section is the information available? (Reagents and Tools Table, Materials and Methods, Figures, Data Availability Section)
Studies involving **human participants**: State details of **authority granting ethics approval** (IRB or equivalent committee(s), provide reference number for approval.	Not Applicable	
Studies involving **human participants**: Include a statement confirming that **informed consent** was obtained from all subjects and that the experiments conformed to the principles set out in the WMA Declaration of Helsinki and the Department of Health and Human Services Belmont Report.	Not Applicable	
Studies involving **human participants:** For publication of **patient photos**, include a statement confirming that consent to publish was obtained.	Not Applicable	
Studies involving experimental **animals**: State details of **authority granting ethics approval** (IRB or equivalent committee(s), provide reference number for approval. Include a statement of compliance with ethical regulations.	Not Applicable	
Studies involving **specimen and field samples:** State if relevant **permits** obtained, provide details of authority approving study; if none were required, explain why.	Not Applicable	

Dual Use Research of Concern (DURC)	Information included in the manuscript?	In which section is the information available? (Reagents and Tools Table, Materials and Methods, Figures, Data Availability Section)
Could your study fall under dual use research restrictions? Please check biosecurity documents and list of select agents and toxins (CDC): https://www.selectagents.gov/sat/list.htm	Not Applicable	
If you used a select agent, is the security level of the lab appropriate and reported in the manuscript?	Not Applicable	
If a study is subject to dual use research of concern regulations, is the name of the **authority granting approval and reference number** for the regulatory approval provided in the manuscript?	Not Applicable	

Reporting

The MDAR framework recommends adoption of discipline-specific guidelines, established and endorsed through community initiatives. Journals have their own policy about requiring specific guidelines and recommendations to complement MDAR.

Adherence to community standards	Information included in the manuscript?	In which section is the information available? (Reagents and Tools Table, Materials and Methods, Figures, Data Availability Section)
State if relevant guidelines or checklists (e.g., **ICMJE, MIBBI, ARRIVE, PRISMA**) have been followed or provided.	Not Applicable	
For **tumor marker prognostic studies,** we recommend that you follow the **REMARK** reporting guidelines (see link list at top right). See author guidelines, under 'Reporting Guidelines'. Please confirm you have followed these guidelines.	Not Applicable	
For **phase II and III randomized controlled trials**, please refer to the **CONSORT** flow diagram (see link list at top right) and submit the CONSORT checklist (see link list at top right) with your submission. See author guidelines, under 'Reporting Guidelines'. Please confirm you have submitted this list.	Not Applicable	

Data Availability

Data availability	Information included in the manuscript?	In which section is the information available? (Reagents and Tools Table, Materials and Methods, Figures, Data Availability Section)
Have **primary datasets** been deposited according to the journal's guidelines (see 'Data Deposition' section) and the respective accession numbers provided in the Data Availability Section?	Yes	Data Availability
Were **human clinical and genomic datasets** deposited in a public access-controlled repository in accordance to ethical obligations to the patients and to the applicable consent agreement?	Not Applicable	
Are **computational models** that are central and integral to a study available without restrictions in a machine-readable form? Were the relevant accession numbers or links provided?	Not Applicable	
If publicly available data were reused, provide the respective **data citations in the reference list**.	Not Applicable	
